# Stricture of the Urethra

**Published:** 1855-05

**Authors:** 


Stricture of the Urethra.—This disease attracts considerable attention in the hospitals of Paris, and more especially has it done so within the last few years, during which time many important points in its pathology and treatment have been elucidated. Since the clear establishment of the point that organic strictures depend not upon a lesion of the mucous membrane of the canal, but upon the effusion and subsequent contraction of organizable fibrin in the corpus spongiosum, throughout the whole or a part of its thickness, the means for their relief have become at once more rational, and more efficient. In all cases dilatation by means of caoutchouc or wax bougies is first tried, and of course in a large majority of cases succeeds. The instrument is not allowed to remain in the canal, but is removed in from two ten minutes; one of larger size being each day employed until the desired dilation is produced. In cases which resist this treatment, incision from within outward, after the method of Reybard is adopted; and, in some few cases, incision through the entire body of the corpus spongiosum from without inward is practiced; cauterization, as advised by Prof. Lallemand, is now universally rejected, and on all sides I have heard the assertion that after cauterization the stricture is always rendered more callous and difficult of cure than it was before.

In 1853, M. Reybard, who long ago introduced the internal section of strictures, published a work on the subject, and proposed an instrument consisting of a sound, bearing within it a concealed blade, which being introduced into the canal, the blade is protruded, and an incision into the corpus spongiosum effected, the work gained for him the prize of Argenteriel of 12,000 francs. Like all other innovations, the process has had, and still has, many detractors, and it certainly cannot be without danger, when we bear in mind the manifold risks from cutting into a venous plexus such as the corpus spongiosum; the proportion of deaths has, however, been very small, and out of numerous cases in which I have seen the method employed by M. Civiale during the past year, I cannot recall a single one which has terminated fatally. M. C. employs an instrument of his own invention, which is regarded as superior to that proposed by Reybard. As I have already stated, the operation of Syme, of Edinburg, consisting of an incision from one, to one and a half inches in length, through the corpus spongiosum, from without inward, upon a grooved sound in the urethra, is sometimes employed; thus M. Adolphe Richard has practiced it six times, and M. Sédillot four times—in Great Britain it appears to have gained many partisans. Mr. Syme has, since the beginning of 1844, performed it seventy times, McKenzie seven times, and Ferguson four times; while others, whose names are unknown to me, have brought the number to one hundred and fifteen. Out of Mr. Syme's seventy cases not one died; M. Richard out of six lost one; and, out of the entire number, only five deaths are reported—in each instance the fatal termination has been produced by purulent absorption. Previous to operating after Syme's method, M. Nélation, the distinguished surgeon of the Hospital of the Clinique, some days since, made some remarks on the subject, in which he expressed himself as not satisfied with the reliability of the statistics of this operation, and regarded it as much more dangerous than they would lead us to believe. During the year 1854, the operation was several times performed by Dr. Carnochan, surgeon of Ward's Island Hospital, New York, with entire success; but, as I possess no detailed notes of the cases, I can enter into none of their particulars.

As I am aware that these views are at variance with those long and generally received in America, I will state some of the authorities upon which I make, what may be otherwise considered, too bold an assertion; at the same time I will refrain from going at length into the subject, as its great interest and importance make me desirous of devoting to it, at some future time, a separate article. Having experienced some difficulty in determining the present views of the following gentlemen, from their published works, and being desirous of avoiding even a chance of error, I called upon each of them, and was kindly furnished with the following information:—

M. Aug. Mercier informed me that although he does not deny the *possibility* of constriction of the canal at the prostatic portion, from great destruction and subsequent reparation of the prostate gland, he has never met with a case in his practice; and, in the membraneous portion, without disease of the bulb, (whose extremity extending under the commencement of the former may cause error,) he denies the possibility.

M. Malgaigne stated that, if strictures occur beyond the spongy portion of the canal, they must be very rare, for in his extensive practice he has never met with one in the prostatic portion, and the only time that he has been led to the belief that they could exist in the membraneous, was in a post mortem examination, when he found a constriction in that part, which appeared to him to resemble strictures in the spongy portion; this is the only case that he, in a very large experience, and paying particular attention to the point, has ever seen, and he regards the numerous cases reported as due to errors in measurement, which it is well known are sometimes ludicrously great.

M. Caudmont, a clinical lecturer of this city, who has for years made a speciality of this branch of surgery, denies, in a manner the most positive, the possibility of organic stricture below that portion of the urethra in contact with the corpus spongiosum. It is, however, upon the opinion of M. Civiale that I would lay most stress, and, at another time, I shall report it fully; although he does not state the point clearly in his work, he informed me not two days since, that he does not admit the existence of organic structure in any part of the urethra below the bulb; there is, says he, a state of muscular contraction which occurs in the membraneous portion when the bulbous is affected by stricture, and this has led to numerous errors. M. Ammussat, as quoted by M. Mercier, agrees with the opinion of Civiale.

Among the many subjects which offer themselves for investigation in Paris, there is none whose study is attended with more interest and profit than that of syphilis; each year's researches appear to be rewarded by new and important facts in its history, and, at last, it is being classified by clear and determinate rules, though it is true that these are, as yet, too recent to have overcome the opposition of those who, until of late, have been blind to their existence. Two grand classes of chancres have for years past been admitted, namely: the simple chancre and the specific or indurated; the first, is a purely local affection, and whether treated or not, whether heroically attacked with mercury or left to itself, will get well without affecting the constitution with syphilis. The second variety, or the specific, is always, unless checked in its earliest infancy, followed by constitutional affection, and when once fairly fixed in the system, can only be relieved, and not cured by our art. The simple variety is known by the following well marked characteristics:—First, it is multiple; second, its border is not indurated; and, third, it gives a bubo which suppurates; and the specific is no less distinct in its symptoms—first, it is nearly always single; second, its border is indurated; and, third, it gives induration of the glands of the groin which *never* suppurate. All this is well known to you, but the point at which I wish to arrive is this:—Ricord believes that these two classes arise from the same poison, which is modified in its action by the constitution of the individual; thus if five men are exposed to the same contagion, three will contract specific chancres, while the other two will contract only simple ones, which all, under no circumstances, affect the constitution.

In opposition to these views, let me cite those of M. Clerc, an intelligent and conscientious investigator of this city; I give them from notes on his clinical lectures. M. C. believes that there exist two separate and distinct viruses, one of which, if successfully inoculated, will always produce a non-infecting chancre, and the other always an infecting one; in a person never having had constitutional syphilis, this will be the invariable result, the one kind no more producing the other, than the inoculation of small pox in an unprotected person will produce varioloid. Suppose, however, that the individual has already had constitutional syphilis, the predisposition is destroyed, and the inoculation, even of specific matter, will thereafter produce only a simple chancre, as in one protected by vaccine, the inoculation of variola will only produce varioloid. In his researches, M. Clerc follows this plan:—A patient presenting himself at his clinique, with a specific or non-specific chancre, as the case may be, he immediately takes the name and residence of the female who has communicated it, and arming himself with the presence of the police, he searches her out, and discovers the kind of chancre which she has. The results have, invariably, been in consonance with the fact, that chancres produce their like, the rule being no where broken, except where exemption has been purchased at the price of previous constitutional affection. To the specific variety he gives the name chancre, while to the other he applied that of chancroid. In support of the assertion, that to a constitution fortified by previous syphilitic affection, the inoculation of specific matter imparts only a non-specific sore, the extensive and well known experiments of Ricord appear; and there are, besides, numerous other instances on record. At the clinique of M. Clerc, there visits one of his friends, a physician who has had constitutional syphilis; he does not hesitate to allow M. C. to inoculate him with the pus of specific chancres, and all the trials that they have made only produced a simple chancre, which soon gets well, and leaves no effect on the constitution.

In my last, I mentioned to you the experiments of M. Bazin, on Porrigo favosa, and his views as regards its vegetable origin, at the same time stating my inability to say in what light they were regarded by the profession. His success has been remarkable, and his views appear very generally admitted. Favus, Mentagra, and probably Herpes tonsurant, he also regards as due to a vegetable parasite, and, with slight modification, applies to them the same treatment. It appears that for a length of time a religious sect of France, called the "Frères Mahon," have possessed, and culpably kept secret, for their own aggrandizement, an effectual cure for this class of diseases, and it is highly probable that the physician of "St. Louis" has, by scientific researches, arrived at the same happy results, which they did by expiricism; actuated, however, by a more truly christian spirit, he has published his means of success freely to the world.

Before closing this letter, allow me to mention a fact of some interest in a pathological point of view, though probably in itself of no practical importance, on account of the rareness of its occurrence. The fact is one to which my attention was called in the service of M. Blache, at "Hôpital des Enfants Malades," namely: a paralysis of the veil of the palate, occurring after inflammation of the fauces, especially when that inflammation is of diphtheritic character. When the patient is in a state of convalescence, there is suddenly observed great difficulty of deglutition, more especially of liquids, which at each attempt regurgitate by the nostrils; at the same time there is dfficulty in pronunciation, and much to the anxiety of friends, the patient loses the power of expressing many words, while other words have that peculiar and disagreeable sound observed in persons suffering from cleft palate. The power of inflating the cheeks and of blowing is lost, and that which renders the affection grave in children. the act of suction is impaired, and sometimes lost. Examination of the palate shows traces of inflammation, redness and tumefaction existing; its contractile power is lost, and sensibility to such degree impaired, that a needle can be passed through the part without causing pain, and cauterization with nitrate of silver practiced without giving rise to any unpleasant sensation. Contractibility, under the influence of electricity, however, still exists, and the passage of a current shows distinct agitation and contraction in the veil and pillars. The prognosis is ordinarily so favorable, and the treatment so nearly negative, that the mere interest of the fact, as a pathological one, must pardon its perhaps useless insertion here, the more especially as my endeavor has hitherto been to confine my remarks to points of practical importance.—*Correspondence of the Charleston Medical Journal.*

